# Characterization of the first chloroplast genome of *Tabebuia* (Bignoniaceae)

**DOI:** 10.1080/23802359.2020.1791003

**Published:** 2020-07-23

**Authors:** Luiz Henrique M. Fonseca, Lúcia G. Lohmann

**Affiliations:** Departamento de Botânica, Instituto de Biociências, Universidade de São Paulo, São Paulo, Brazil

**Keywords:** Bignoniaceae, chloroplast genome, phylogeny, Chaco

## Abstract

The chloroplast genome of *Tabebuia nodosa* is described and characterized here. This species is endemic to the Chaco and the first species of *Tabebuia* to have its organelle genome sequenced, providing a genomic resource for phylogenetic inferences. The plastome of *T. nodosa* is 158,454 bp in length, with a large single-copy of 85,406 bp, a small single-copy of 12,785 bp, and inverted repeats of 30,116 bp each. It contains 131 genes, with 86 protein-coding genes, 37 tRNA, and 8 rRNA. Overall, the GC content is 38.2%. The *T. nodosa* plastome resembles the structural organization of plastomes commonly found in flowering plants, including those of other genera of Bignoniaceae. A phylogenetic analysis combining a subset of Bignoniaceae plastomes confirms the placement of *T. nodosa* within the *Tabebuia* alliance with maximum support.

*Tabebuia* Gomes ex DC. (Bignoniaceae) is a charismatic genus composed by trees and shrubs (Gentry 1992a; Grose and Olmstead 2007a). The genus currently includes approximately 67 species distributed throughout the Neotropics and centered in Central America and the Great Antilles (Grose and Olmstead 2007b). *Tabebuia* is included within the *Tabebuia* Alliance, which includes conspicuous members of several tropical forests that are often used as ornamentals due to its showy flowers (Gentry 1992a). The only phylogenetic study to date focusing on the *Tabebuia* Alliance sequenced the plastid regions *trn*L-F and *ndh*F of 15 species of *Tabebuia* (Grose and Olmstead 2007a).

*Tabebuia nodosa* (Griseb.) Griseb. is one of the few species of *Tabebuia* that occurs in the Chaco (Grose and Olmstead 2007b). This species is characterized by the reduced leaves (<4 cm in width) and yellow corollas (Gentry 1992b). *T. nodosa* has never been sampled in a phylogenetic study and its placement remains uncertain. Here, we present the complete plastome of *T. nodosa* (NCBI accession number MT447061), the first plastome available for the genus and use this information to provide its phylogenetic placement.

For this study, we selected the specimen *J.M. Silva 4864*, collected in Brazil, Mato Grosso do Sul, Porto Murtinho (long. −57.8824, lat. −21.699) and deposited at the herbarium SPF, from the Universidade de São Paulo (SPF178881). Total genomic DNA (gDNA) was extracted using the Invisorb^®^ Spin Plant Mini Kit (Invitek, Berlin, Germany). Total gDNA was sequenced and the plastome edited and annotated following the steps detailed in Fonseca and Lohmann (2017). In brief, libraries were assembled with insert size of 300 bp and sequenced using pair-end (2 × 100) on an Illumina HiSeq 2000 system (Illumina Inc., San Diego, CA, USA). A total of 12,712,835 paired-end reads were assembled *de novo* using the steps described in the Fast-Plast pipeline (https://github.com/mrmckain/Fast-Plast; McKain and Wilson, unpublished). Annotation was initially conducted using Chlorobox GeSeq (Tillich et al. 2017) and confirmed in Geneious version 7.1.9 (Kearse et al. 2012).

To infer the phylogenetic placement of *T. nodosa* within the Bignoniaceae, we used the 80 plastome genes used to infer the phylogeny of Angiosperms by Li et al. (2019). DNA sequences were aligned in MAFFT version 7.309 (Katoh et al. 2017). Phylogenetic reconstructions were conducted using 17 plastome sequences, representing 14 species of Bignoniaceae and three outgroups. This dataset was complemented with plastid sequences from three Bignoniaceae species obtained from the OneKP Project (Li et al. 2019). Phylogenetic inferences were conducted using maximum likelihood (ML) with RAxML version 8.2.9 (Stamatakis 2014) and Bayesian criteria (BC) with MrBayes version 3.2 (Ronquist et al. 2012). Branch support was estimated for ML using 1000 bootstrap replicates (bs) and for BC using posterior probabilities (pp).

The *T. nodosa* plastome recovered has the typical quadripartite structure, including a single monomer of 158,454 bp in length, with a large single-copy region of 85,406 bp and a small single-copy region of 12,785 bp, separated by two inverted repeat regions of 30,116 bp each. The genome contains 131 genes, including 86 protein-coding genes, 37 tRNA, and 8 rRNA. The plastome structure, gene content, and gene order of *T. nodosa* resembles that of other Bignoniaceae (Fonseca and Lohmann 2017, 2018; Ma et al. 2020). Like several other Bignoniaceae, *T. nodosa* also shows a total inclusion of the gene *ycf*1 in the inverted repeated region (Fonseca and Lohmann 2017, 2018; Ma et al. 2020). The overall GC content is 38.2%, while the GC content of the individual plastome regions is 36.5% for the LSC, 33.4% for the SSC, and 41.7% for each of the IR regions.

The topologies inferred by ML and BC are identical and concordant with the currently available phylogeny of the Bignoniaceae (Olmstead et al. 2009). *Tabebuia nodosa* is strongly supported (bs = 100, pp = 1) as sister to a clade composed of *Handroanthus umbellatus* (Sond.) Mattos and *Crescentia cujete* L. (Figure 1). This topology corroborates the informally named *Tabebuia* Alliance clade, which includes *Crescentia*, *Handroanthus*, *Tabebuia*, and eight other genera (Olmstead et al. 2009). This phylogeny also corroborates the narrower circumscription of *Tabebuia*, excluding *Handroanthus* (Grose and Olmstead 2007a; Olmstead et al. 2009).

**Figure 1.**
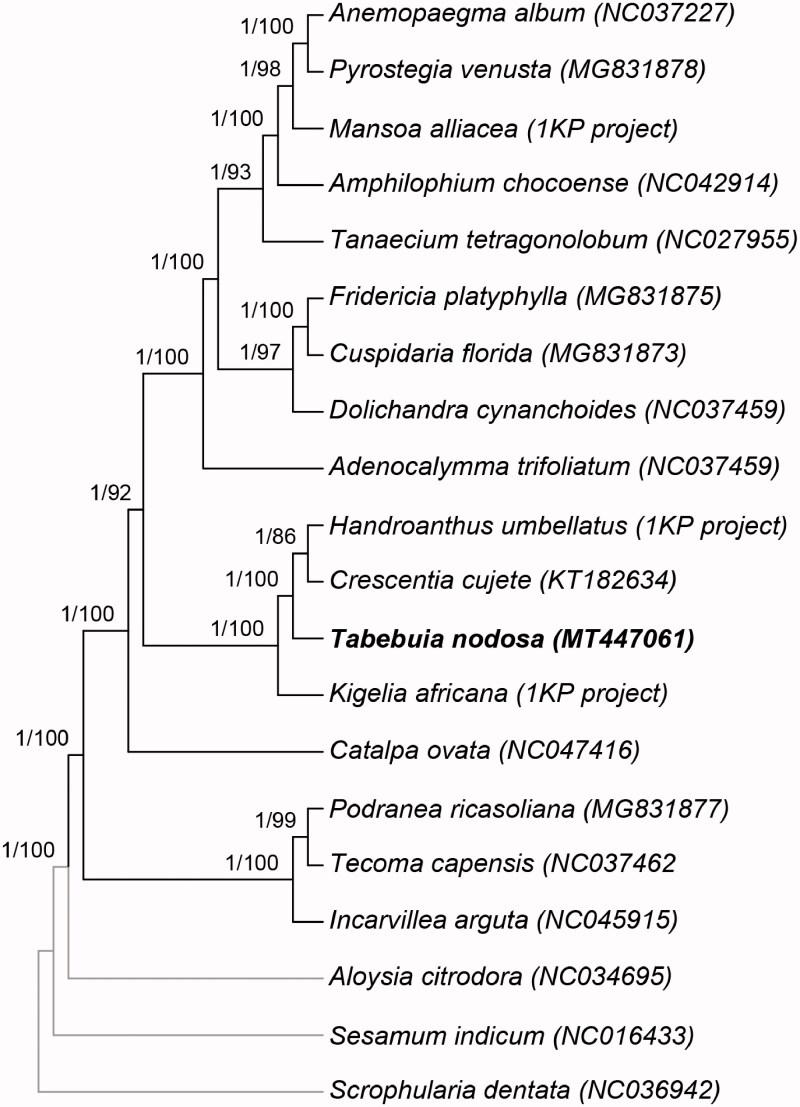
Bayesian mcc tree derived from the analysis of 80 plastid genes. Values above branches denote posterior probabilities and bootstrap values, respectively.

## Data Availability

The data that support the findings of this study are available in GenBank of NCBI at https://www.ncbi.nlm.nih.gov, reference number MT447061.
